# Application of Inspiratory Muscle Training to Improve Physical Tolerance in Older Patients with Ischemic Heart Failure

**DOI:** 10.3390/ijerph182312441

**Published:** 2021-11-26

**Authors:** Monika Piotrowska, Paulina Okrzymowska, Wojciech Kucharski, Krystyna Rożek-Piechura

**Affiliations:** 1 Department of Physiotherapy in Internal Disease, Wroclaw University of Health and Sport, Al. I.J. Paderewskiego 35, Building P4, 51-612 Wrocław, Poland; monika.piotrowska@awf.wroc.pl (M.P.); krystyna.rozek-piechura@awf.wroc.pl (K.R.-P.); 2 Department of Human Biology, Wroclaw University of Health and Sport Sciences, Al. I.J. Paderewskiego 35, Building P4, 51-612 Wrocław, Poland; wojciech.kucharski@awf.wroc.pl; 3 Cardiac Rehabilitation Unit, Hospital of Vratislavia Medica, Saint John Paul II, ul. Lekarska 1, 51-134 Wrocław, Poland

**Keywords:** cardiovascular rehabilitation, heart failure, heart rate, control respiratory system diagnostic techniques, breathing exercises, exercise tolerance

## Abstract

Regardless of the management regime for heart failure (HF), there is strong evidence supporting the early implementation of exercise-based cardiac rehabilitation (CR). Respiratory therapy is considered to be an integral part of such secondary prevention protocols. The aim of the study was to evaluate the effect of inspiratory muscle training (IMT) on exercise tolerance and the functional parameters of the respiratory system in patients with heart failure involved in cardiac rehabilitation. The study included 90 patients with HF who took part in the second-stage 8-week cycle of cardiac rehabilitation (CR). They were randomly divided into three groups: Group I underwent CR and IMT; Group II only CR; and patients in Group III underwent only the IMT. Before and after the 8-week cycle, participants were assessed for exercise tolerance and the functional parameters of respiratory muscle strength. Significant statistical improvement concerned the majority of the hemodynamic parameters, lung function parameters, and respiratory muscle strength in the first group. Moreover, the enhancement in the exercise tolerance in the CR + IMT group was accompanied by a negligible change in the HR_peak_. The results confirm that the addition of IMT to the standard rehabilitation process of patients with heart failure can increase the therapeutic effect while influencing some of the parameters measured by exercise electrocardiography and respiratory function.

## 1. Introduction

Heart failure (HF) is a clinical syndrome caused by structural and functional defects of the heart muscle. These changes cause an impairment of ventricular filling or blood ejection [1]. HF is major public health concern with substantial morbidity and mortality [2]. The use of cardiac rehabilitation (CR) can effectively improve the aerobic capacity and general health of patients with HF. Interestingly, successful CR is a valuable nonpharmacological intervention for patients with HF. It should be a multidisciplinary approach, involving supervised exercise, patient counseling, education, and nutritional counseling. All these components can also improve the quality of life [3].

One of the most common causes of HF is myocardial ischemia induced by myocardial infarction (MI), which is one of the most widely known and dramatic clinical manifestations of coronary artery disease. The symptoms are quite diverse, and the underlying pathogenesis of MI is varied. However, it can be characterized as an interruption in, or an insufficient supply of, oxygen and nutrients on the basis of the current myocardial metabolic demand, most frequently caused by a coronary occlusion. Knowledge of the pathogenesis of myocardial infarction has evolved and allowed for new treatment strategies that significantly improve survival [4]. HR is a multifactorial and systemic disease. Changes are activated at the structural, neurohumoral, cellular, and molecular levels. At the same time, these complex processes lead to excessive volume overload, increased sympathetic activity, and circulatory redistribution. This results in a variety of clinical symptoms [5].

Coronary artery disease, primarily by acute MI, is the most common type of cardio-vascular disease and the leading cause of death in Europe in individuals under 75 years of age [6].

The cellular changes in heart failure include myocyte hypertrophy, abnormalities in calcium homeostasis, excitation–contraction coupling, cross-bridge cycling, and changes in the cytoskeletal architecture [7].

Regardless of the management regime and pharmacological approach, there is strong evidence supporting the early implementation of exercise-based cardiac rehabilitation (CR). This intervention has shown numerous benefits, including improvements in functional capacity and quality of life, and a reduction in the totals for cardiac mortality and morbidity. Physical training can be an effective and reliable alternative for treating patients with heart failure. Rehabilitation can result in an improvement in the cardiovascular risk factors, functional capacity, and health-related quality of life in cardiac patients. [8,9,10]. For this reason, the role of CR has significantly increased over recent years, with clinicians suggesting it be treated as a coequal with pharmacotherapy in the treatment of MI patients. Pharmacology can influence the effectiveness of cardiac rehabilitation, improve cardiac function, and reduce the incidence of adverse cardiac events [11,12]. 

Among the many components of a CR program, respiratory therapy is considered to be an integral part of such secondary prevention protocols. It contributes to an improved prognosis and a reduction in acute or chronic respiratory system complications. Clinical practice guidelines commonly recommend some form of respiratory physiotherapy. One modality that has been the subject of recent investigation is inspiratory muscle training (IMT), with evidence finding it improves functional capacity and inspiratory muscle strength in congestive heart failure (CHF) patients, while also attenuating dyspnea [13]. Current knowledge indicates that inspiratory muscle training is beneficial for improving respiratory muscle strength, functional capacity, and dyspnea in patients with stable heart failure. Additional benefits can also include increased inspiratory endurance and an improved quality of life. The routine screening of patients with HF for inspiratory muscle weakness is recommended [14].

The aim of the study was to evaluate the effect of inspiratory muscle training on exercise tolerance and the functional parameters of the respiratory system in patients with heart failure who are involved in cardiac rehabilitation.

## 2. Materials and Methods

### 2.1. Trial Design

The study was conducted at the “Creator” Rehabilitation Medical Center in Wrocław, Poland, and at the Diagnostics Laboratory of the Department of Physiotherapy in Internal Disease at the University of Physical Education in Wrocław, Poland. An initial sample of 145 potentially eligible heart failure patients, due to myocardial ischemia induced by myocardial infarction, were recruited meeting the following inclusion criteria: age range, 61–75 years; clinically stable cardiovascular status; nonsmoking; no later than 4 weeks following hospital discharge; and no less than 1-year prior involvement in a rehabilitation program. Exclusion criteria were as follows: respiratory disease characterized by a significant reduction in respiratory capacity; aortic and/or iliac artery surgery; signs of vascular damage in the central nervous system; injury of the spine or pelvis; hormone treatments; or a mental disorder mitigating cooperation or patient contact. These studies were simple randomized controlled trials. In order to prepare the randomization list, the appropriate computer-prepared number distribution tables were used.

The Consolidated Standards for Reporting Trials (CONSORT) checklist of 2010 was used to evaluate the quality of reporting [15].

The study was conducted according to the guidelines of the Declaration of Helsinki and was approved by the Institutional Review Board (or Ethics Committee) of The Australian New Zealand Clinical Trials Registry (ANZCTR) (ACTRN12618001871235 and date of approval, 16 November 2018). In addition, a statement indicating that all participants signed the informed consent form prior to their inclusion in this study was added.

### 2.2. Participants

**Figure 1:**
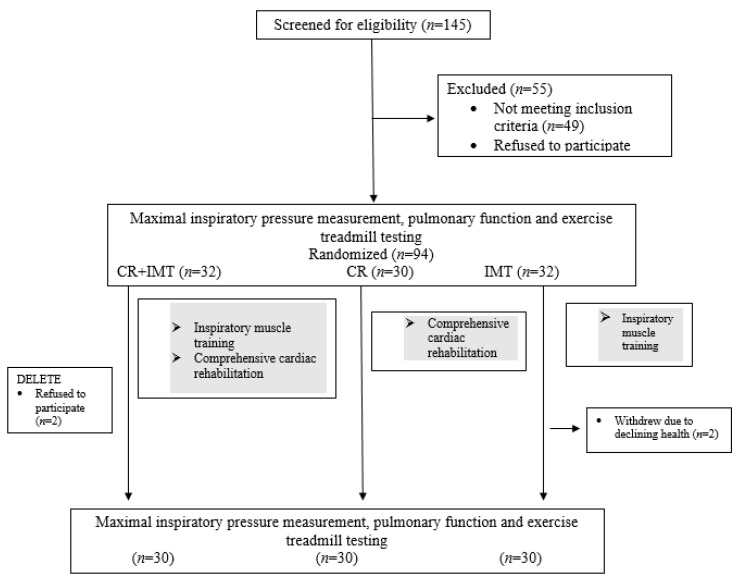
Design and flow of participants through the study.

Following screening for inclusion and exclusion and confirmation for eligibility by a physician, the baseline sample included 94 patients. During the course of the study, 4 individuals discontinued participation; hence, the study only describes the final sample (*n* = 90, 40 females and 50 males) (Figure 1).

### 2.3. Intervention

Cardiac rehabilitation in the CR + IMT and CR groups involved five 45-min training sessions per week. General fitness exercises and resistance training took place twice a week, and interval training was conducted on an ER900 ergometer (Ergoline GmbH, Bitz, Germany) three times a week. General fitness exercises involved group equilibrium, stretching, inspiratory and expiratory exercises, as well as relaxation. Elements of endurance training, which included 8–10 types of endurance exercises involving various muscle groups, were incorporated in the indoor training. (Each set of exercises was repeated 12 to 15 times). The same sets of exercises were done by all patients, and all procedures were performed in accordance with Polish Cardiac Society recommendations [16].

Interval training involved 4-min cycling bouts, with increasing intensity for the first 2 min, and then subsequently decreasing after reaching peak intensity. Before each training session, the patient warmed up on a no-load ergometer. Efforts during cycling training were interspersed with 2-min rests (load 0–5 W). The intensity was initially 40–70% of the preworkout electrocardiographic test. It was then increased by no more than 10 in every 12 training sessions. During training on the ergometer, at the end and beginning of each interval, the heart rates and blood pressures of the patients were measured. The session ended with a 3-min cooling down without workload. Perceived exertion did not exceed 13 on the Borg scale.

Inspiratory muscle training in the CR + IMT and IMT groups was performed five times per week, using the patient’s own personal Threshold IMT device (Appendix A) (Respironics, Chichester, UK). Four sessions were performed at home, and one was supervised by a physiotherapist at the Creator Center. A familiarization session was held in which the participants were instructed on how to correctly use the device. Patients had to perform the training standing up and using a nose clip. Ensuring a tight seal around the mouthpiece, the inspiratory phase involved a rapid, energetic, diaphragmatic inhalation. The expiratory phase was to be slow and prolonged until residual volume was reached, as each subsequent inhalation was to start at this point [17,18].

The inspiratory loads on the Threshold IMT were individually determined on the basis of the maximal inspiratory pressure (PI_max_) obtained during the pulmonary function test (detailed below). This measure, expressed in kPa, was converted to cmH_2_O using the formula, 1 kPa = 10.2 cm H_2_O, in order to adjust the Threshold IMT. The inspiratory load in the first week of training was limited to 30% of the PI_max_ and two 5-min sessions per training day to prevent overtraining [19]. In the succeeding weeks, the loads and durations were progressively increased according to a predefined protocol (Table 1).

### 2.4. Outcome Measures

Primary outcomes were assessed by pre- and postintervention comparisons of the tolerance to exercise via exercise electrocardiography on a treadmill, of pulmonary function via spirometry, and of inspiratory muscle function by maximal inspiratory pressure. 

### 2.5. Exercise Electrocardiography 

In all patients in the groups, the tests were carried out twice, at the beginning of the experiment and after its completion (after 8 weeks). The ECG test was performed on the treadmill (Challenger, Aspel, Zabrzów, Poland, Cardio ECG system for exercise testing, Perfekt MD Rozinn Electronics, New York, NY, USA) according to the recommendations of the Polish Cardiac Society. The patient was prepared for the test, was calm, and did not make any efforts on the day of the examination. EKG tests were done in the morning, at the right temperature, and in the large and ventilated exercise testing laboratory. The study was conducted using the modified Bruce protocol, and the stress test was limited to symptoms. The patient finished the test because of subjective exhaustion, which did not allow further examination. Each patient achieved at least 70% of the maximum heart rate. After the test, the patient was observed by the medical staff until the subject was completely calmed down. This study used the difference in the exercise tolerances (MET: metabolic equivalent) measured during the initial and final tests. The baseline test measures were also used to quantify which exercise module (A, B, or C—rehabilitation models depended on the level of exercise tolerance) the participant should complete [20].

### 2.6. Inspiratory Muscle Strength 

The PI_max_ was measured while the patient was seated using the same Flowscreen spirometer, with the addition of a special accessory. All procedures were performed in accordance with ATS/ERS guidelines (2002). Verbal encouragement was provided in order to motivate the participant to perform a maximal forced inhalation for 2–3 s. This procedure was repeated in order to obtain 5 to 10 (depending on the participant’s ability) valid measurements. The three highest measures (difference below 5%, or 5 cm H_2_O) were averaged to determine the PI_max_. The baseline PI_max_ was used to calculate the inspiratory load applied during the IMT [21].

### 2.7. Pulmonary Function Testing

Pulmonary function was assessed using an ambulatory spirometer (Flowscreen models 780 and 578, ver. 1.3, Jaeger, Germany). This validated and reliable instrument was used to determine the vital capacity (VC), forced vital capacity (FVC), forced expiratory volume in 1 s (FEV_1_), and forced expiratory volume in 1 s as a % of the vital capacity (FEV_1_%VC) [21].

### 2.8. Statistical Analysis

Basic descriptive statistics were used to calculate the arithmetic mean, standard deviation, minimum and maximum values, median with upper and lower quartiles, range, and skewness. The distribution of the dataset in each group was screened for normality using the Shapiro–Wilk test. Testing confirmed the assumption of normality and the parametric tests were performed. Comparisons between the group means for anthropometry were performed with one-way analysis of variance (ANOVA). The group × time interaction for all outcome measures was assessed by repeated measures ANOVA, with Fisher’s least significant difference (LSD) post hoc.

## 3. Results

### 3.1. Participant Characteristics

The mean (SD) characteristics of this sample were: female age, 63.2 (9.1) years; male age, 61.7 (10.3) years; female height, 1.62 (0.19) m; male height, 1.78 (0.13) m; female mass, 85.09 (12.73) kg; and male mass, 89.2 (13.45) kg. All patients were NYHA class I and II. Patients with left ventricular ejection fraction with an average value of 54. All declared themselves to be nonsmokers and free of respiratory disease. The participants were equally (*n* = 30, 12 females and 18 males) randomized by a pregenerated list among three 8-week treatment strategies involving outpatient CR and/or IMT with a specific fitness module. The first group received CR concurrent with IMT (CR + IMT), the second group CR only, and the third group IMT only. Most of patients were post percutaneous coronary intervention (PCI) (Table 2).

### 3.2. Exercise Electrocardiography

Noteworthy is the fact that the enhancement in the exercise tolerance in the CR + IMT group was accompanied by a negligible change in the HRpeak (Table 3).

### 3.3. Inspiratory Muscle Strength

The postintervention PI_max_ [l] and PI_max_ [%] significantly increased in the CR + IMT and IMT groups, whereas the PI_max_ [kPa] significantly increased in the CR group (Table 4).

### 3.4. Pulmonary Function

A significant increase after the intervention was observed in IMT for the VC [1] and the FEV_1_ [%]. Moreover, in this group, a significant decrease in the VC [%] and the FEV_1_ [1] was observed. In the CR + IMT group, a statistically significant reduction was observed among the analyzed pulmonary variables. In the CR group, significant increases in the VC [%], the FEV_1_ [1] and the FEV_1_ [%] parameters were observed. On the other hand, the VC [1] decreased significantly (Table 5).

## 4. Discussion

In our study, we observed significantly statistical improvements concerning the majority of the hemodynamic parameters, lung function parameters, and respiratory muscle strength in the first group. Interestingly, we also obtained a reduction in the chronotropic response during the exercise of the patients.

A review of the available literature finds that among the many benefits of physical activity and exercise is a reduction in mortality and morbidity from cardiovascular disease. Meta-analyses indicate that exercise training is most strongly linked to reduced mortality [22]. These same systematic reviews accentuate that the training modality, exercise intensity, weekly training frequency, and session duration are of critical importance when prescribing a physical activity program for this population.

The results of this study regarding the increased peak METs with the change in the HR_max_ can potentially be interpreted as the improvement in the stroke volume during exercise, or the heart rate control for a higher exercise intensity. El Missiri et al. (2020) also show a significant change in the HR after cardiac rehabilitation. At the end of the 6-week program, they achieved a significant increase in the METs achieved and the exercise time. They did not obtain a change in the peak heart rate. By contrast, at the end of the 12-week program, there was a significant increase in the METs, exercise times, and peak heart rates from the baseline [23].

Laoutaris et al. (2004) evaluated the benefits of inspiratory muscle training (IMT) in patients with chronic heart failure (CHF). In the study group, the resting heart rate was significantly reduced during training (77 +/− 3.3 versus 80 +/− 3 beats/min, *p* < 0.05). The maximum inspiratory pressure also increased. Perceived dyspnea, as assessed by the Borg scale, decreased on both the treadmill and while walking. Exercise testing and quality of life were also improved. In contrast, the PI_max_ significantly increased in the control group, but no significant effects on the exercise capacity, heart rate, dyspnea, or quality of life were observed. It is worth noting that the resting heart rate decreased significantly, and the peak heart rate tended to decrease in the training group during the treadmill tests. The authors speculate that it is possible that the training-induced bradycardia achieved the same workload with a lower demand for sympathetic circulation, heart rate, and oxygen in the myocardium. Thus, it is possible to increase the reserve exercise capacity. These findings may reflect a beneficial feedback between improvements in respiratory function and shifts in the autonomic balance from sympathetic to vagal prominence and/or the involvement of a diaphragmatic-mediated reflex, which may influence the overall sympathetic tone [24].

The investigation of respiratory muscle performance indicates that the exercise-induced fatigue of the inspiratory muscles limits exercise capacity [25,26,27,28]. Yet, there is scientific evidence indicating that even short intensive breathing exercises have a significant influence on improving the health status of cardiovascular disease patients [29]. As patients with cardiovascular disease or CHF may have reduced inspiratory muscle strength, this type of training may contribute to enhanced exercise tolerance and, therefore, improved prognosis. 

At the same time, it is worth pointing out that excessive metaboreflex of the respiratory muscles in patients with HF may be one of the reasons for exercise intolerance. It has been shown that the use of an individually adjusted IMT can reduce this response [30].

In patients with HF, excessive metaboreflex manifests as an increase in the vascular resistance at the systemic level, which limits cardiac output, stroke volume, and O2 supply and generates impaired exercise capacity and peak VO2 [31].

Research by Moreno et al. confirms that IMT alleviates the magnitude of the changes in the oxygen saturation in the intercostal muscles and the forearm during respiratory fatigue and, thus, reduces the metaboreflex [32].

The present study confirmed this observation, in which various exercise protocols combined with IMT resulted in significant increases in the VC, FEV_1_, PEF, and MIF after an 8-week intervention. These effects were not observed in the group performing CR only (no IMT). The meta-analysis performed signals an improvement in the cardiovascular parameters after IMT, although there is high variability in the results and further high-quality research is required [14].

The current state of knowledge indicates that respiratory muscle fatigue, observed by the change in the MIP (maximal inspiratory pressure) before and during the exercise test, significantly correlates with the exercise capacities of patients with cardiovascular diseases [33]. The available literature indicates that patients with CHF with inspiratory muscle weakness show greater gains in the 6-min walking distance and peak oxygen consumption compared to those with the normative maximum inspiratory pressure [34]. In our study, we demonstrated that the inspiratory muscle strength is significantly reduced and remains at the level, PI_max_: 30–40%. Ribiero et al., conducting studies evaluating respiratory muscle strength in patients with HF, found a reduction in inspiratory muscle strength and indicated that it is an important prognostic indicator for patients with heart failure [34]. The available literature indicates that patients with CHF with inspiratory muscle weakness show greater gains in the 6-min walking distance and peak oxygen consumption compared to those with the normative maximum inspiratory pressure [35]. In addition, the available analyses indicate that inspiratory muscle training is beneficial for improving respiratory muscle strength, functional capacity, and dyspnea in patients with stable heart failure and respiratory muscle weakness [14]. It is also interesting that, as observed in other reports, the use of IMT may be helpful to people with respiratory muscle weakness who, because of their health condition, are unable to perform conventional exercise. The current state of knowledge indicates that isolated IMT may increase the inspiratory muscle strength, functional capacity, and quality of life in patients with heart failure. IMT in conjunction with a subsequent intervention may only produce a slight increase in inspiratory force. Isolated IMT with a higher load may be considered a complementary intervention, especially for those who do not adhere to conventional rehabilitation and who have respiratory muscle weakness [36].

The available systematic reviews show that the training effects on HF patients are heterogeneous and varied. On the basis of this analysis, IMT was found to improve the functional capacity of patients with HF [37].

Bosnak-Gluclu et al. (2010) also administered a respiratory muscle training intervention to CHF patients, at an intensity of 40% of the PI_max_, and registered significant increases in the FEV_1_, FVC, and PEF [38]. Forgiarini et al. (2007) also introduced a similar training protocol in CHF patients, at 30%of the PI_max_, but did not observe any significant changes in the ventilatory function. These and other discrepancies between the IMT protocols suggest that higher exercise intensity is a prerequisite for stimulating the enhanced ventilator function [39]. This finding is, in part, confirmed by the results of the present study, in which we adopted a more vigorous intensity of 60% of the PI_max_.

We observed an increase in inspiratory muscle strength only in the groups which received IMT. These, and other adaptations of respiratory muscle training, were also obtained in other treatment groups, including athletes, healthy individuals, and patients with respiratory, cardiovascular, or neuromuscular diseases [40,41,42,43,44,45].

The available literature also finds that improved respiratory muscle strength and endurance following IMT may also affect other functions. Laoutaris et al. (2004) report a decrease in the resting heart rate with a concurrent increase in oxygen consumption (VO_2_) in CHF patients after a 10-week IMT intervention at 60% of the PI_max_ [46]. Similar findings, with regards to the VO2, were reported by Mancini et al. (1995) for a similar population [47]. However, the results of our study did not show similar effects on the HR_peak_ and exercise tolerance via measurement of the MET in the group receiving only IMT. Instead, significant improvements in these variables were observed only in the CR + IMT and CR groups.

Worthy of mention is an investigation similar to the present study, although involving patients with bronchiectasis [48]. This sample received 8 weeks of pulmonary rehabilitation (PR), or PR with IMT. While a significant improvement in the exercise capacity (VO_2_) and endurance was observed in both the PR and PR + IMT groups, inspiratory muscle strength increased only in the PR + IMT group. Such a finding was also observed herein, in that the exercise tolerance was significantly stimulated in the CR and CR + IMT groups, but inspiratory muscle strength increased only in the CR + IMT group.

At the same time, the available literature lacks studies assessing changes in the respiratory parameters with the use of IMT in cardiac patients. In our study, a statistically significant reduction in the VC [1] was demonstrated in the CR + IMT group and in the CR group. It was only in the IMT group that a significant increase in this parameter was obtained. VC changes may indicate a change in the compliance of the chest wall. It is hypothesized that the reduction in VC may be evidence of a decreased susceptibility [49]. In our study, a statistically significant increase in the FEV1 was obtained in the CR group and the IMT group [%]. The current state of knowledge indicates that the increase in the FEV1 is focused on reducing airway resistance and increasing lung flexibility. In addition, it is also believed that participants may become more aware of their breathing patterns [50].

On the basis of these physiological and functional benefits, respiratory muscle training (inspiratory and/or expiratory) has seen growing popularity, particularly as a form of active exercise. This training modality has been strongly recommended in the rehabilitation of patients with respiratory, neuromuscular, and/or cardiovascular disorders because of the numerous practical considerations [42,51,52,53]. It has been found to be an effective, low-cost, and easily administered intervention, involving a short training time, which was noted by the participants in the present study and has been emphasized by other researchers [54].

The limitation of this study may be the absence of a direct assessment of the physical performance using a spiroergometric test. Our study shows the measurement exercise tolerance by indirect examination on a treadmill. Therefore, we believe that the study should be continued and conducted with CPET consideration. A good level of (normal) baseline measurements of the patients’ spirometric parameters may be a limiting factor for the increase in these variables after IMT.

## 5. Conclusions

The combination of traditional cardiac rehabilitation and inspiratory muscle training influenced the increased peak METs, with the change in the HR_max_ potentially interpreted as the improvement in the stroke volume during exercise, or the heart rate control for higher exercise intensity. This may be important in patients with chronic heart failure.

After the applied inspiratory muscle training and cardiac rehabilitation in patients, a significant increase in the PI_max_ was observed, with a simultaneous increase in the lung ventilation parameters compared to the group with only standard cardiac rehabilitation.

The results confirm the need for the standard rehabilitation of all patients with heart failure, and the addition of IMT increases the therapeutic effect while improving the function of the respiratory system.

## Figures and Tables

**Table 1:** Description of 8-week IMT intervention.

Week	1	2	3	4	5	6	7	8
Inspiratory load [%PI_max_]	30	40	40	50	50	60	60	60
Session duration [min]	2 × 5	2 × 8	2 × 11	2 × 11	2 × 13	2 × 13	2 × 13	2 × 15

**Table 2:** Baseline participant characteristics (excluding withdrawn patients) with *p* values for intergroup comparisons; data presented as mean (SD), except for sex and NYHA class.

	Total	CR + IMT	CR	IMT	*p*
Age [years]	62.77 (8.05)	61.03 (7.46)	63.60 (5.1)	63.67 (7.59)	0.36
Males [*n* (%)]	54 (60)	18 (60)	18 (60)	18 (60)	
Mass [kg]	84.41 (9.91)	82.66 (11.39)	86.03 (9.21)	84.53 (8.97)	0.43
Height [m]	1.75 (0.09)	1.74 (0.08)	1.76 (0.08)	1.73 (0.09)	0.16
BMI [kg/m^2^]	27.76 (3.02)	27.54 (2.84)	27.68 (3.26)	28.06 (3.03)	0.79
NYHA class [*n* (%)]					
I	46 (51)	13 (43)	15 (50)	18 (60)	0.70
II	44 (49)	17 (57)	15 (50)	12 (40)	0.57
Intervention [*n* (%)]					
PCI	46 (51.11)	12 (40)	17 (56.66)	17 (56.66)	0.55
CABG	44 (48.88)	16 (53.33)	13 (43.33)	15 (50)	0.71
Drugs [*n* (%)]					
Beta-blockers	82 (91.1)	26 (86.67)	27 (90)	29 (96.67)	0.89
Statins/fibrates	65 (72.22)	23 (76.66)	21 (70)	21 (70)	0.88
Duretics	19 (21.1)	3 (10)	7 (23.33)	9 (30)	0.55
Cardiovascular risk factors [*n*(%)]					
Hypertension	49 (54.44)	16 (53.33)	17 (56.66)	16 (53.33)	0.78
Lipid disorders	54 (60)	17 (56.67)	16 (53.33)	21 (70)	0.66

Abbreviations: Values are presented as means (standard deviation). Significant differences in bold and with an asterisk. *p* < 0.05; BMI—Body Mass Index; NYHA class—New York Heart Association Functional Classification; PCI—percutaneous coronary intervention; CABG—coronary artery bypass graft; LVEF—left ventricular ejection fraction.

**Table 3:** Comparison of selected data on HRs, test durations, and MET pre- and postinterventions. Group × time interaction effects for exercise electrocardiography variables by repeated measures ANOVA, with Fisher’s least significant difference (LSD) post hoc (significant differences in bold and with an asterisk).

Variable		CR + IMT	CR	IMT
**HR_peak_** **[bpm]**	Preintervention	117.73 ± 12.55	115.57 ± 13.63	115.47 ± 14.03
	Postintervention	120.23 ± 15.14	117.80 ± 15.96	117.73 ± 14
	*p*	**0.97**	0.02 *	0.26
**Test duration [min]**	Preintervention	10.80 ± 2.21	10.07 ± 2.26	10.96 ± 3.2
	Postintervention	12.48 ± 1.89	11.84 ± 2.83	11.61 ± 2.68
	*p*	0.00 *	0.00 *	0.02 *
**MET**	Preintervention	6.82 ± 2.79	6.61 ± 2.34	7.62 ± 3.6
	Postintervention	8.74 ± 2.4	8.17 ± 3.12	8.10 ± 3.5
	*p*	0.00 *	0.00 *	0.11

Abbreviations: * *p* < 0.05; HR—heart rate; MET—metabolic equivalent.

**Table 4:** Comparison of selected data on the PI_max_. Group × time interaction effects for absolute and relative PI_max_ (significant differences in bold and with an asterisk).

Variable		CR + IMT	CR	IMT
PImax [kPa]	Preintervention	2.82 ± 1.03	2.95 ± 1.35	3.02 ± 1.27
	Postintervention	6.02 ± 2.25	3.59 ± 2.26	5.69 ± 1.85
	*p*	0.00 *	0.05 *	0.00 *
PImax [%]	Preintervention	35.20 ± 14.87	37.39 ± 16.55	38.32 ± 16.92
	Postintervention	76.02 ± 26.73	44.17 ± 24.5	70.83 ± 21.88
	*p*	0.00 *	0.06	0.00 *

Abbreviations: * *p* < 0.05; PI_max_—maximal inspiratory pressures.

**Table 5:** Pre- and postintervention pulmonary function variables. Data presented as mean (SD) and standard deviations.

Variable	CR + IMT		CR		IMT		*p*
	Pre-Intervention	Post-Intervention	Pre-Intervention	Post-Intervention	Pre-Intervention	Post-Intervention	
VC [l]	3.95 ± 0.96	3.82 ± 0.93	3.64 ± 0.76	3.42 ± 0.96	3.95 ± 1	5.82 ± 0.99	0.00 *
VC [%]	95.30 ± 15	93.10 ± 14.34	94.20 ± 19.54	95.40 ± 18.84	93.10 ± 12.85	94.20 ± 12.76	0.00 *
FEV_1_ [l]	3.40 ± 0.83	3.05 ± 0.84	3.30 ± 0.9	4.10 ± 0.8	3.40 ± 0.79	3.30 ± 0.97	0.00 *
FEV_1_ [%]	93.04 ± 19.71	91.10 ± 15.97	93.01 ± 20.28	100.02 ± 18.9	94.20 ± 15.37	94.90 ± 16.26	0.02 *

Abbreviations: Values are presented as mean. * *p* < 0.05; VC—vital capacity, FEV_1_—forced expiratory volume in first second.

## Data Availability

Data are stored in a deidentified state and can be made available by reasonable and appropriate request.

## Appendix A. Practical Applications

Research indicates, after adding inspiratory training to standard rehabilitation, the improvement in the lung ventilation parameters and inspiratory muscle forces, as a consequence of which improved effort tolerance occurred. Therefore, it would be useful to introduce inspiratory muscle training for cardiac rehabilitation. This is all the more beneficial because the Threshold IMT device (Respironics, Chichester, UK) is very easy to use and practical and does not require large amounts of money.
